# Cost and Effectiveness of Fiber-Reinforced Polymer Solutions for the Large-Scale Mitigation of Seismic Risk in Reinforced Concrete Buildings

**DOI:** 10.3390/polym13172962

**Published:** 2021-08-31

**Authors:** Ciro Del Vecchio, Marco Di Ludovico, Andrea Prota

**Affiliations:** 1 Department of Engineering, University of Sannio, Piazza Roma 21, 82100 Benevento, Italy; 2 Department of Structures for Engineering and Architecture, University of Napoli Federico II, Via Claudio 21, 80125 Napoli, Italy; diludovi@unina.it (M.D.L.); aprota@unina.it (A.P.)

**Keywords:** seismic retrofit, shear failure, FRP, beam-column joints, costs, local strengthening, global retrofit

## Abstract

Recent seismic events have demonstrated that the high vulnerability of existing reinforced concrete (RC) buildings is mainly due to a lack of proper seismic detailing and the employment of poor-quality concrete. The reconstruction process following the 2009 L’Aquila earthquake highlighted that strengthening these buildings using solutions based on fiber-reinforced polymers (FRPs) can be both efficient and cost-effective. Indeed, their light weight, ease of installation, and the availability of specific guidelines and standards strongly supported their use in design practices, where they were the strengthening technique employed the most. This paper analyses and discusses the data on the actual cost and effectiveness of FRP solutions for seismic strengthening of existing RC buildings. To this end, the large database relating to the L’Aquila reconstruction process was used to select 130 RC buildings strengthened with FRP systems or FRPs combined with other techniques. Details of direct costs, including at the member level, and the types and percentages of strengthened members are analysed for both local and global strategies. This study thus provides readers with valuable data for use in cost-benefit analyses of FRP systems schemes to mitigate seismic risk at large-scale.

## 1. Introduction

Most reinforced concrete (RC) buildings in the Mediterranean region were built in the last century and designed using old seismic provisions. They therefore lack any proper seismic detailing, making them extremely vulnerable to seismic action. In this context, field reconnaissance after the 2009 L’Aquila earthquake has highlighted the poor performance of beam-column joints and short columns, which often failed in shear due to the lack of transverse shear reinforcements [1,2]. Experimental tests and analytical studies have demonstrated that fiber-reinforced polymers (FRPs) can be an effective strengthening solution that significantly improves seismic performance [3]. Much of this research revealed that wrapping joint panels with FRPs significantly increased their shear capacity [4,5,6,7,8,9,10]. Other studies at the building level also outlined the effectiveness of properly designed FRP solutions at improving the global seismic capability of existing RC structures [11,12,13,14]. Furthermore, the reduced disruption caused during their implementation, as well as the material’s light weight, ease of installation and durability, led to the strong endorsement of FRPs as an efficient and cost-effective strengthening solution [15,16,17].

**Figure 1:**
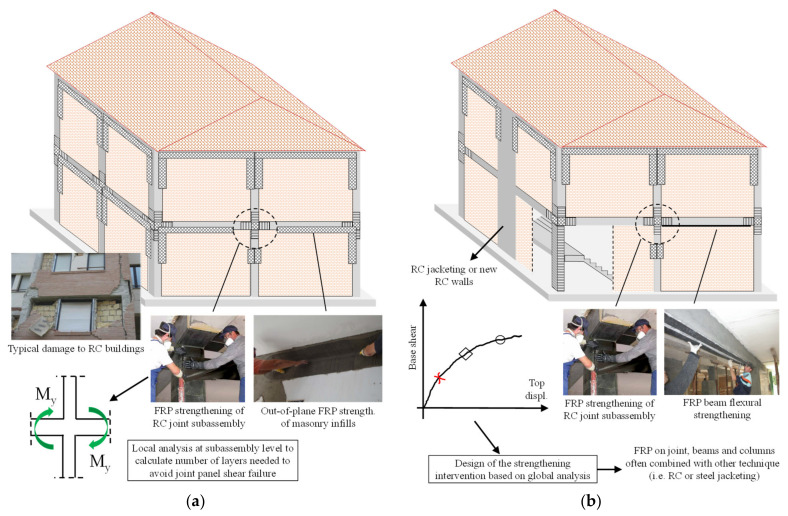
Strengthening strategies adopted during the L’Aquila reconstruction process: Local strengthening of exterior RC beam-column joints and infills to prevent out-of-plane failure (**a**); Global strengthening (**b**).

FRP systems can be used to improve the building seismic performance by following different strengthening strategies: local strengthening or global retrofitting (see Figure 1). Available studies, guidelines, and national standards [15,18,19,20] emphasise that, to prevent local failures, repairs and/or local strengthening should recover/enhance the seismic capacity (strength or ductility) of a building’s most critical structural and non-structural components, which should be achieved without significantly changing global structural behaviour (i.e., the global stiffness and mass). FRP systems can be very effective at improving the performance of RC buildings by preventing both local shear failures at the beam-column-joint or short-column levels and the overturning of exterior infill walls [21] (see Figure 1a). This approach (i.e., local strengthening) does not usually require a global structural analysis, with calculations at the member level instead used to determine the target shear demand and quantify the enhancement capacity. Conversely, global retrofitting requires the analysis of an entire structural system to identify the main structural and non-structural weaknesses. The strengthening solution is then tailored towards meeting a fixed performance standard, e.g., the life-safety limit state (LSLS). The building code in Italy [20], for example, enables the latter to be quantified using the safety index score at the LSLS, ζ_E_, expressed as the capacity/demand ratio for the peak ground acceleration (PGA_C_/PGA_D_) at the reference site. Experimental tests and numerical studies have highlighted that premature shear failures commonly limit the displacement capacity of existing structural systems (see Figure 1b). FRP systems can be a viable solution for enhancing the shear strength of the critical members promoting the more ductile failure modes associated with a larger displacement capacity (see Figure 1b). However, these techniques cannot resolve major structural deficiencies, such as those related to a building’s irregularities and/or lack of stiffness, or modify the plastic failure mechanism. Consequently, in these cases, they are often used in conjunction with traditional and more invasive retrofit solutions (e.g., RC walls, RC jacketing, steel bracing).

These strengthening techniques, along with major pre-code and code-formulation activities, led to the production of various guidelines and standards [19,21,22], enabling the use of these novel solutions in design practices. This was a significant endorsement of FRP systems as seismic-strengthening solutions for buildings and infrastructures.

In 2009, the Abruzzo region in Italy was hit by a major earthquake (M_w_ = 6.3), with the epicentre in the municipality of L’Aquila. This caused a number of fatalities and huge economic losses [23]. A massive reconstruction process followed on from the emergency phase. This was mainly supported with government funding that covered repair costs in their entirety. Funds for seismic strengthening were also made available. The earthquake provided a unique opportunity to collect technical, social, and economic data on the damage caused to existing buildings, as well as on how they were repaired and strengthened. This produced a large database of 5775 records [24] that are still in use today, largely among the scientific community. The reconstruction process distinguished two phases, with the first concerning “lightly damaged” buildings [24] and the second those that sustained “heavy damage” [24]. The classification of the damage was foregrounded on the outcomes of the usability assessments conducted in the emergency phase with the AeDES form [25]. “Light damage” involved buildings rated B or C based on the form’s categorisations (i.e., B = a totally or partially unusable building, but usable after short-term countermeasures; C = partially unusable), but also those with an E-rating (i.e., an unusable building) where there was only slight damage to structural members but diffuse damage to non-structural elements, namely E-B buildings in the reconstruction process. These lightly damaged RC buildings (n. 1961) were mainly subjected to repair or local-strengthening interventions. This comprised repairing the damage to structural and non-structural components (including finishes) and locally strengthening RC members and infill-frame connections to prevent out-of-plane overturning. An economic threshold for seismic strengthening was fixed at 150 €/m^2^ (of the overall gross surface area), including VAT and the fees of design and technical practitioners.

In contrast, the heavily damaged RC buildings (n. 554; E-rated structures that were not demolished but repaired and retrofitted) were subjected to global retrofitting (designed by the practitioners engaged by owners for the reconstruction process). This comprised classic and innovative solutions like RC jacketing, steel jacketing, FRP jacketing, new RC walls, steel bracings, and base isolation. These repair costs were also covered in their entirety, with the threshold set at 400 €/m^2^ for the strengthening required to achieve a safety index, ζ_E_, between 0.6 and 0.8 (unless private funding was also available).

Although the employment of FRP materials in seismic retrofits of existing buildings is widespread, simple tools for quickly selecting the best strategy and evaluating the costs of any interventions are still required. Indeed, such tools are currently unavailable in the literature, meaning that assessments of initial costs based on price lists may lead to significant approximations of actual costs. This is due to the difficulty of predicting any required complementary actions and the variability of building-to-building construction standards. As a consequence, studies based on actual data may help researchers and designers to determine the benefits and cost of FRP-strengthening interventions on a large scale. This paper therefore concerns the use of FRPs in the seismic strengthening of existing RC buildings. In particular, the data collected during the L’Aquila reconstruction process are analysed to provide insights on the different strengthening solutions and related benefits. Actual costs on strengthening is assessed and simple tools are proposed to enable practitioners to produce quick estimates of the costs of any proposed retrofit interventions. The study therefore provides readers with useful data for performing cost-benefit analyses on the large-scale application of FRP systems in seismic-risk mitigation strategies.

## 2. Repair Costs at the Building Level

**Figure 2:**
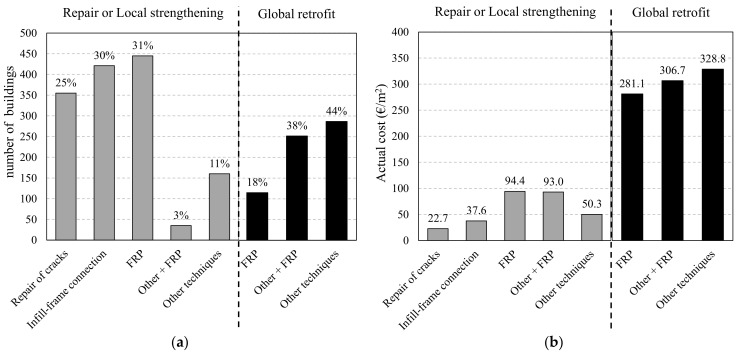
Distribution of strengthening interventions (**a**) and actual costs (**b**) for buildings subjected to repair or local strengthening (1416 buildings) or to a global retrofitting (454 buildings).

An initial analysis was conducted using the L’Aquila-region database referred to earlier, which contains information on 1416 lightly damaged and 454 heavily damaged RC buildings subjected to repairs and local strengthening or global retrofitting, respectively. In particular, the data (where available) were employed to identify the techniques adopted and the real costs of the work. Figure 2a demonstrates that FRP systems were used in 31% of the RC buildings subjected to repairs/local strengthening and 18% of those undergoing global retrofit. These percentages increase to 34 and 56%, respectively, considering also the buildings strengthened with FRPs combined with other techniques (i.e., Other + FRP). A significant portion of the buildings in the first category (30%) also underwent infill-frame connection interventions, which commonly use FRP-based solutions. Consequently, it is clear that strengthening strategies which employed FRPs in some forms were those adopted the most to conduct effective and fast repairs and improve seismic performance.

The actual costs, expressed in terms of €/m^2^ (of the overall gross-surface area), to implement these strengthening techniques, are reported in Figure 2b. These were obtained from technical documents submitted by designers to the technical and financial committee established by the Italian government to oversee funding requests [23]. The quotes were reviewed and, if necessary, amended before approval. The mean costs for the strengthened buildings were as follows: lightly damaged where only repairs were undertaken—approx. 22.7 €/m^2^; strengthening of the infill-frame connection alone to prevent out-of-plane overturning—approx. 37.6 €/m^2^; locally strengthened with FRP solutions—approx. 94.4 €/m^2^; FRP schemes combined with other techniques—93.0 €/m^2^; and other, non-FRP solutions—approx. 50.3 €/m^2^, which is significantly less than for FRP-based techniques. This is because this final category mainly includes buildings requiring only the replacement of a few damaged RC members. All of the costs described above include charges for the design and technical assistance of practitioners, but not VAT.

In relation to global strengthening, the actual costs were: FRP solutions—approx. 281.1 €/m^2^; FRP systems combined with other techniques—approx. 306.7 €/m^2^; and other techniques alone—approx. 328.8 €/m^2^. These costs are significantly higher than those associated with local-strengthening solutions. This is not only because detailed global analyses were required to assess the seismic performance of these buildings, but also because more extensive interventions were undertaken with the aim of achieving a minimum safety index score of 0.6. More details and the distribution of the strengthening costs at the building level can be found in Di Ludovico et al. [23,24].

### Database Definition for the Component Level Analysis

**Figure 3:**
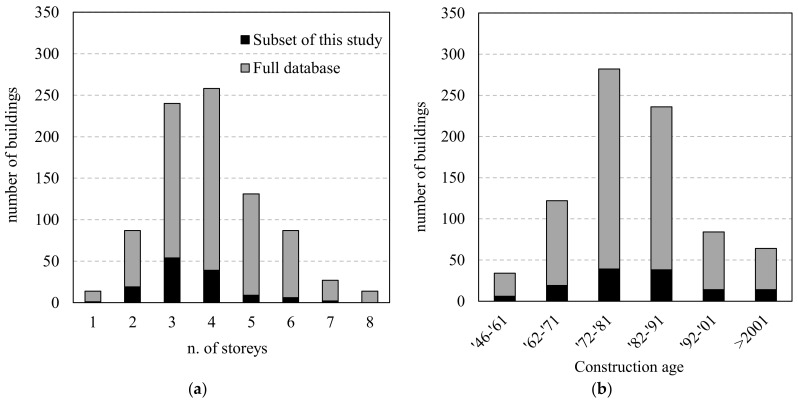
Distribution of the complete database of FRP-strengthened buildings (FRP and Other + FRP) (858 buildings) and the subset used in this study (130) in relation to: n. of storeys (**a**); construction age (**b**).

A subset of 130 RC buildings, representing about 7.2% of the overall population, was extracted from the complete database of 1870 (lightly and severely damaged) structures for which repair costs were available. This was used to conduct a detailed investigation of the FRP-based strengthening solutions, the types of application and their effectiveness, the distribution of the strengthened members, and the actual costs. The decision to limit the dataset to 130 buildings (7.2% of the complete database) was due to the significant effort required to collect and analyse the strengthening at the component level. Indeed, our examination of more than 130 estimates of repair costs was a time-consuming process, as they included the costs of all the materials and activities required to apply the planned repair and strengthening solution. The selected buildings were extracted randomly by matching the frequency distributions of the complete database in relation to the construction age and the number of storeys (see Figure 3).

Figure 3b shows that most of the buildings in the database were built in the period 1962–1991 and, as a result, were designed with now obsolete seismic-design provisions (Ricci et al., 2011). They prescribed limitation to number of storeys and provided dimensions of beam and column sections and the least amount of steel reinforcement required. However, it should be noted that L’Aquila has been classified as a seismic zone since 1915 (R.D.L. 573, 1915), which should be subjected to careful consideration when attempting to generalise the results of this study to other regions. Figure 3a shows that most of the buildings in the reference subset have three or four storeys.

**Figure 4:**
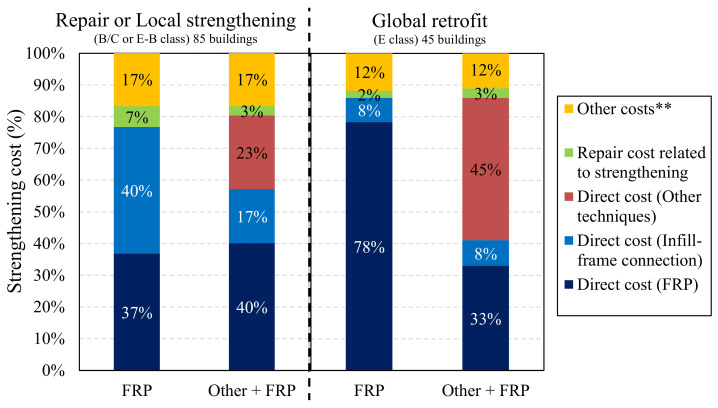
Distribution of the strengthening costs for different categories. (** This includes general costs for installing the construction field, safety measures, and professional fees related to strengthening actions.).

Our initial discussion of these actual costs is oriented towards the percentage distributions of the total retrofit costs reported in Figure 2. The results are shown in Figure 4 with reference to buildings strengthened by means of FRP schemes alone or FRP + other techniques; FRP solutions designed according to a repair/local-strengthening or global-retrofit strategy are also analysed.

The direct costs associated with local FRP-strengthening interventions for improving the capacity of RC members were about 37% of the total cost. Most of this cost (about 40%) involved the direct cost of installing infill-to-frame connections using composite materials, generally fibre-reinforced cementitious mortar (FRCM). The other 60% comprised repair costs related to strengthening interventions, as well as other general costs (e.g., construction-field installation, safety measures) and professional fees, with the latter two categories amounting to 7 and 17% of the total strengthening cost, respectively. In the buildings strengthened using other techniques combined with FRP systems, the cost of the former amounted to approx. 23% of the total strengthening costs and the latter about 40%. In these instances, the direct cost of installing infill-to-frame connections was about 17%, which is significantly less than the expenditure on the buildings retrofitted using FRP systems alone. It should, however, be noted that this infilling solution was employed in just a few cases in this subset of buildings strengthened using a combination of other techniques and FRPs.

In the heavily damaged RC buildings that underwent global retrofitting using FRP systems, most of the overall expenditure comprised the direct cost of these systems (about 78%), with only 8% related to the installation of infill-to-frame connections. The cost of strengthening interventions amounted to about 2% of the total, which is relatively low given the level of disruption associated with global strengthening. However, it is worth noting that the extent of the damage in these severely damaged buildings (those subjected to global retrofit solutions) may mean that some of these repair costs were included in the general expenditure on repairs. Consequently, the numbers in Figure 4 in relation to global retrofitting are undoubtedly underestimates. Nevertheless, we were able to disaggregate the repair cost on repairs [26], which showed that these costs in relation to severely damaged buildings were about 81.26 €/m^2^, amounting to around 15.34% and 26% of the total reconstruction costs and strengthening costs, respectively. The other cost was approximately 12%, which is slightly less than that observed for locally-strengthened buildings. This is probably because some costs, such as those related to the installation of the construction field, are less significant in percentage terms in these cases.

In relation to the buildings strengthened using other techniques combined with FRP systems, the total cost was about 9% more than that observed for the buildings strengthened using FRP interventions alone (see Figure 2b). However, the direct cost of the strengthening solutions in percentage terms was about the same as in the FRP-strengthened buildings. In particular, 33% was associated with FRP strengthening, 8% with infill-to-structure connections, and 45% with implementing other strengthening techniques. The reduction in the direct cost of the FRP strengthening was due to the need to use other strengthening solutions to resolve major structural weaknesses.

**Figure 5:**
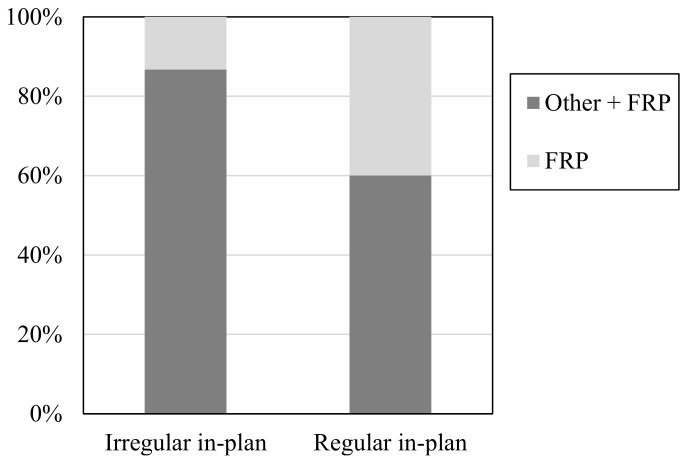
Use of FRPs and other techniques for regular and irregular in-plan buildings.

It is worth noting that where the strengthening intervention has to be designed using the results of a global analysis, FRP systems alone are unable to resolve all of the structural weaknesses detected (e.g., soft storeys, significant torsional effects). Accordingly, it is necessary to also adopt other strengthening techniques that can increase lateral stiffness. Indeed, as shown in Figure 5, the percentage of buildings strengthened using a combination of FRPs and other techniques increased for those with an in-plan irregularity (defined according to EC8 [27] prescriptions).

## 3. Adopted FRP-Strengthening Interventions

In order to focus on the FRP solutions adopted in L’Aquila, what follows is a detailed analysis of the subset of buildings strengthened with either FRPs alone in a local-strengthening strategy (58 buildings) or global retrofits (16 buildings). In particular, the percentage distribution of the adopted FRP solutions and the direct cost for each structural member as well as the mean number of FRP layers and strengthened members for each building are analysed and discussed. The selection of these parameters takes into account the fact that a designer involved in the retrofitting of an existing building may need to know the type and mean number of RC members to be strengthened, as well as the number of FRP layers required. This will enable the production of preliminary estimates of the cost of the interventions, their location, and the associated level of disruption.

### 3.1. Locally Strengthened Buildings

**Figure 6:**
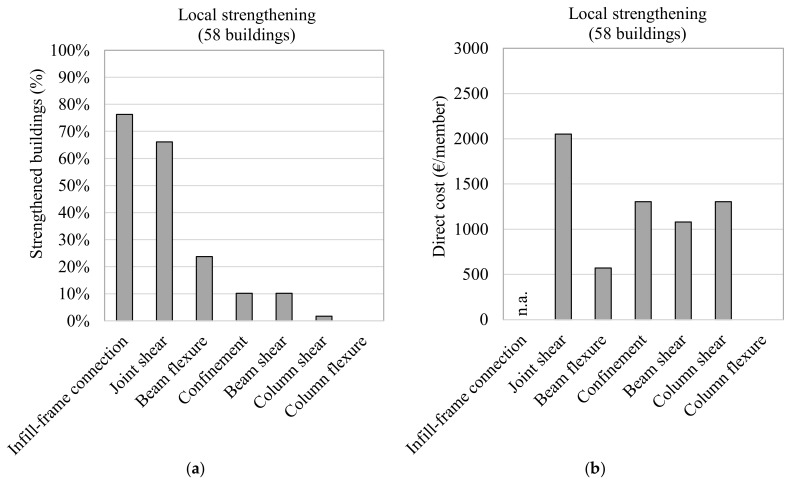
FRP-strengthening interventions adopted in a local-strengthening strategy (**a**,**b**).

The goal of preventing local failures without significantly changing global structural behaviour (i.e., global stiffness and mass of the building) means that the main scope of a local-strengthening intervention is to recover/enhance the seismic capacity (strength or ductility) of the most critical structural and non-structural components. The distribution of the interventions adopted in a local-strengthening strategy are reported in Figure 6 in terms of the percentage of buildings involved (Figure 6a) and the mean direct cost of each member, expressed in €/member (Figure 6). The data are also summarised in Table 1 in relation to: the overall number of buildings; the percentage of buildings that employed the strengthening intervention; the mean number of FRP layers used for strengthening; the direct cost, expressed in €/member and normalised for the demolition and reconstruction (taken as 5344 €, 4008 €, and 6012 € for the beam-column joints, columns and beams, respectively [28]); and the mean number of strengthened members.

The data shows that, in most of the buildings under investigation, the designer chose to improve the infill-frame connection (77.6% of cases) and increase the seismic capacity of beam-column joints (67.2%), as indicated in the ReLUIS guidelines [21]. The former was achieved with one layer of FRCM containing composite or steel fibres. The direct cost per member and the mean number of strengthened members are unavailable, because the estimates in the database report the extension in terms of the linear metre of the intervention.

The reinforcement layout described in the ReLUIS guidelines and reported schematically in Figure 9a was often employed to strengthen the unconfined beam-column subassemblies. This process involves: using the top column’s shear strength to resist the infill action (done with a uniaxial steel fabric); shear strengthening the joint panel (commonly with a quadriaxial fabric); confining the ends of the columns to improve the deformation capacity; and shear strengthening at the end of the beam (typically using uniaxial FRP fabrics). The mean number of layers employed to enhance joint panel shear strength was about 1.6. The direct cost of such a reinforcement strategy was around 2051.5 €/member (38.4% RC) and the mean number of strengthened joint subassemblies in each building was about 15.

Strengthening RC beams to enhance their flexural capacity occurred in 24.1% of buildings, with the application average being 1.2 layers and the mean direct cost about 572.1 €/member (9.5% RC). This technique was used on roughly six beams per building and localised at either the end of the members, in correspondence to cracks, or to recover the capacity of corroded steel bars. The solution was often adopted to resolve original deficiencies that were mainly related to gravity load issues; in some cases, the post-earthquake inspections revealed the development of vertical cracks on beams, probably resulting from the vertical load increase that occurred due to vertical seismic action. Column confinement or beam shear strengthening was utilised in 10% of buildings at an average cost of 1305.6 (32.5% RC) or 1080.1 €/member (18.0% RC), respectively.

### 3.2. Globally Retrofitted Buildings

**Figure 7:**
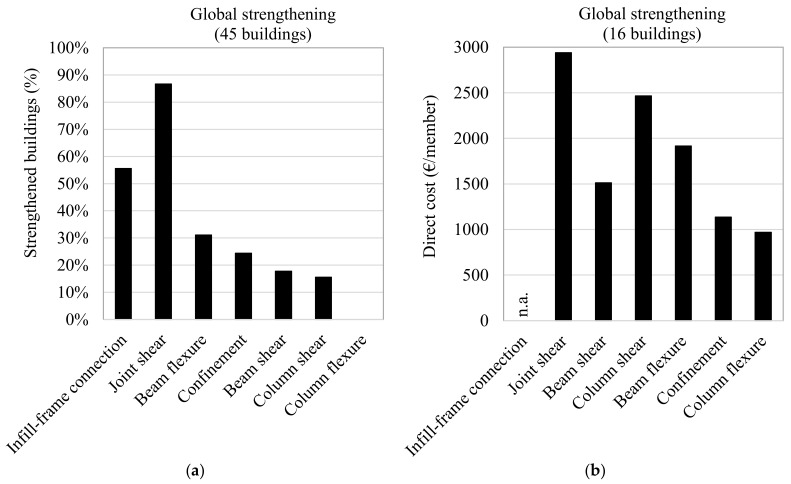
FRP-strengthening interventions adopted in accordance with a global strengthening strategy (**a**,**b**).

Figure 7a,b depict the number of buildings affected (in % terms) and the direct costs (expressed as €/member) of the solutions adopted in the building subset subjected to FRP-strengthening designed in accordance with a global retrofit strategy.

The data show that, in these cases, the solutions used the most were the shear strengthening of the joint subassembly (87.5% of buildings) and the infill-frame connection technique (50% of buildings). The mean number of layers applied was about 2.1 when the joint-panel shear strengthening was designed using the level of demand on the surrounding members, which was identified with a global analysis that had a target safety-index of more than 0.6. This number of layers is higher than that used when a local-strengthening strategy is adopted; this is because a local approach is based on only limited knowledge on joint geometries, reinforcement details and actions. In turn, the mean cost of the joint strengthening rose to 2939.2 €/member (55.0% RC), while the number of strengthened members also increased, to approximately 37.4.

Beam and column shear strengthening was employed in 31.2% of buildings using a mean n. of applied layers of about 2.0 or 1.3, respectively. This aimed to increase the seismic capacity at the end of the beams and on the short columns (e.g., those of the staircase), which are very vulnerable to seismic action. The adoption of a global retrofit strategy to design the beam flexural strengthening required the use of a similar number of layers to that for the local strengthening, but at a higher cost. This is because the FRP strengthening was mainly designed to withstand gravity loads, with fibres applied along a significant length of beam (rather than across the crack).

In relation to column confinement, the results reveal that 1.0 layer of uniaxial fibre was generally enough to significantly increase the plastic hinge rotation at the member ends. Where this strengthening solution was required, most of the columns had to be confined at the top and bottom ends, leading to a rather high mean number of confined columns.

## 4. FRP Strengthening to Improve the Seismic-Risk Class

The Italian government has recently encouraged strategies for mitigating the seismic risk in existing buildings by offering a tax deduction for the costs of seismic strengthening [29]. This is only available if the seismic capacity of a building’s critical structural and non-structural components and, in turn, its overall seismic performance and seismic-risk class, are improved. Local strengthening or global retrofit strategies can be used to this end. The strengthening should aim to both increase seismic safety using interventions on the structural system and reduce expected annual losses (EALs) by protecting non-structural components from damage caused by low-intensity earthquakes [29]. The emphasis is on local strengthening to enable the widespread application of any the strengthening interventions, helping designers to increase seismic capacity by retrofitting the most critical structural components. The guidelines support an improvement of one seismic-risk class if strengthening interventions are applied to all the unconfined perimetral beam-column joints and to the perimetral infills to increase the connection with the structural system. These actions, as widely demonstrated by experimental and analytical studies, improve the building’s seismic performance significantly [12]. Such interventions were also those employed the most in the local and global-strengthening strategies used in existing RC buildings during the L’Aquila reconstruction process.

### Seismic Strengthening of Beam-Column Joints

In the context of improving the seismic performance of beam-column joints, this study proposes a detailed analysis of both the distribution of the joint-panel shear strengthening per building height and its effectiveness in terms of an increase in the global seismic-safety index. The data collected previously on the subset of severely damaged buildings repaired and retrofitted using only FRP interventions designed according to a global-strengthening strategy are processed and discussed. Consequently, although our approach concerns a particular dataset and buildings in a specific region, the analysis may nevertheless produce provisions for more general use on common deficiencies in existing RC structures.

**Figure 8:**
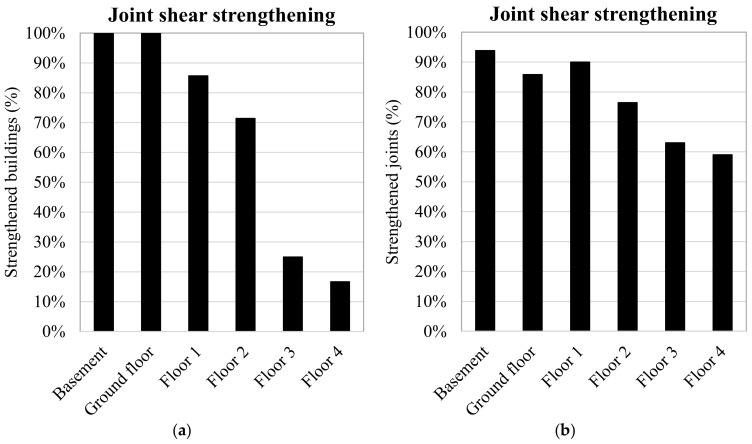
FRP-strengthening interventions adopted in a global strengthening strategy (**a**,**b**).

The results reported in Figure 8 show the distribution of the joint shear strengthening achieved per building height. In particular, this is portrayed in terms of: the percentage of buildings strengthened, which is calculated with respect to the total number of buildings (Figure 8a); and the percentage of strengthened joints, determined in relation to the total number of unconfined joints available on a floor (Figure 8b).

In detail, Figure 8a shows that the percentage of the beam-column joints strengthened with FRPs decreased with each increase in floor number from the ground floor up. All the buildings had been strengthened at the joints of the lower floors (basement and ground floor), while the 86% and 71% of the joints had been strengthened for floors 1 and 2, respectively. These numbers decreased to only 24% and 17% for floors 3 and 4.

**Figure 9:**
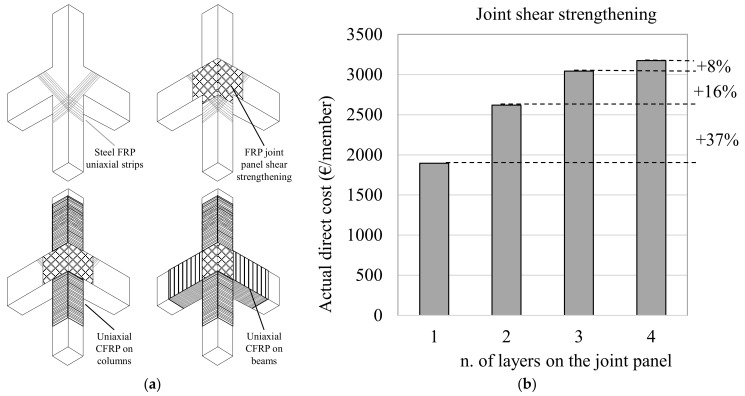
Fabrication steps of the joint subassembly FRP strengthening (**a**); direct cost of FRP shear strengthening of the beam-column joints as a function of the n. of layers applied to the joint panel (**b**).

Meanwhile, Figure 8b shows the number of unconfined joints strengthened with FRPs relative to the total number of joints on a particular floor in a percentage term. On average, the shear strengthening interested the 90% of the joints in the basement and on the ground and first floors. This percentage decreased with height, reaching 60% on floor 4. This occurred because global analyses of the buildings’ performances under lateral loads were carried out to assess the joint-shear demand required to achieve the targeted safety index and this demand often falls on higher floors due to the reduction in the floor shear. Further analyses can provide additional information on the cost of joint panel shear strengthening joint (see Figure 9a as an example of the layout employed the most) and the relationship with the number of FRP layers applied.

Figure 9b shows the rise in the direct cost of installing FRP systems on the joint subassembly as a function of the increase in the number of layers applied. The mean cost of a strengthening scheme with one layer of quadriaxial CFRP is about 1894.3 €/m^2^. This rises to 2619.8 €/m^2^, equating to an increase of approximately 37%, when two layers are used. The percentage rise in cost for joints strengthened with one layer of CFRP falls as the number of layers applied grows (approx. 16% and 8% for three and four layers, respectively).

**Figure 10:**
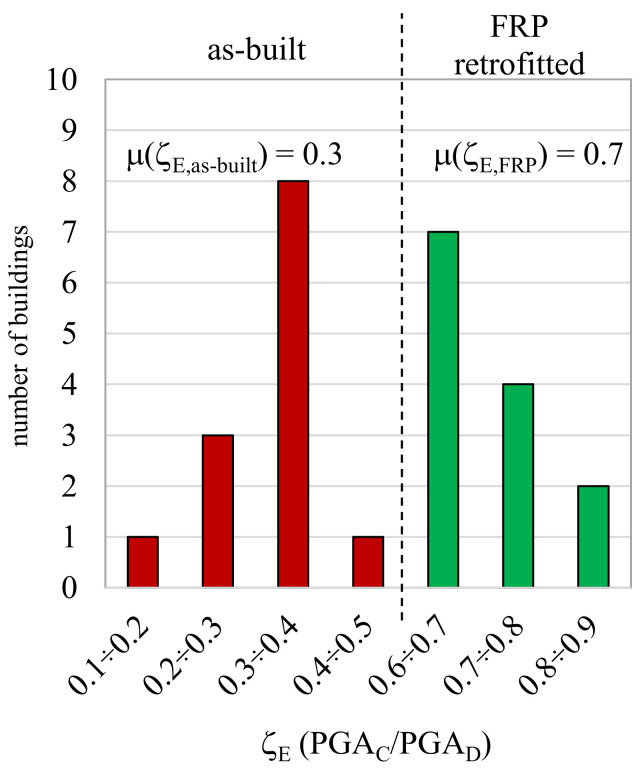
Seismic-safety index at the LSLS for the as-built and FRP-retrofitted configurations.

The use of joint-panel shear strengthening combined with other minor FRP interventions (see Figure 8 and Table 2) led to a significant increase in the overall seismic capacity. This is demonstrated clearly in Figure 10, which compares the building seismic capacity, represented by the safety index, ζ_E_, in the as-built and FRP-strengthened configurations. As can be seen, there was a significant increase in seismic capacity, from a mean of about 0.3 for the as-built structures (ζ_E_,_as-built_) to one of roughly 0.7 for those strengthened with FRPs (ζ_E_,_FRP_).

**Figure 11:**
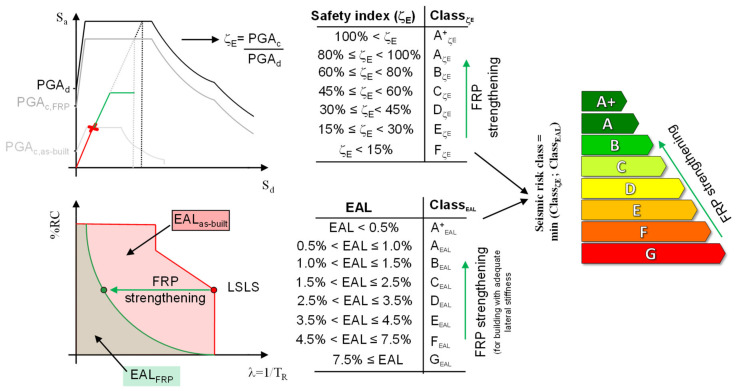
Impact of FRP-strengthening on the seismic-risk class, calculated based on Italian guidelines [29].

Strengthening the most critical structural components with FRPs improved both the global seismic performance (represented, e.g., by the increase in the displacement capacity of a pushover curve; see Figure 11) and the safety index, ζ_E_. Such a rise in the latter at the LSLS may lead to a significant increase in Class_ζE_, based on the classifications set out in the Italian guidelines and summarised in Figure 11. However, a reduction in EALs (increase of Class_EAL_) is also required to achieve an effective increase in the associated seismic-risk class. In structures designed to sustain low seismic actions, with adequate lateral stiffness, which was the case for most of the existing RC buildings in L’Aquila, FRP-strengthening prevents local premature brittle failures (conventionally assumed to be the attainment of the LSLS). This allows the performance at the LSLS to be improved, resulting in a significant drop in EALs (see Figure 10).

It can thus be concluded that, although FRP systems do not increase lateral stiffness and, therefore, cannot directly contain any damage to non-structural components, the improvement in seismic safety at the LSLS may often result in lower EALs and a better seismic-risk class. More detailed studies using life-cycle cost (LCC) methodologies are required to produce global, and more detailed, results on the cost-effectiveness of various FRP-strengthening solutions. This analysis should account for the initial cost of the investment, savings due to the reduction in EALs with respect to as-built configurations, and the costs of repairs/maintenance, reconstruction, and end-of-life [30]. In particular, the possibility of recycling the materials used in the construction [31] should be carefully considered and would enable better assessments of the end-of-life costs associated with FRP solutions.

## 5. Conclusions

This paper examines large-scale seismic-risk mitigation strategies that employ FRP-strengthening systems. In particular, the local and global schemes that can be used to design such solutions are described and discussed with reference to real data on 130 RC buildings collected during the reconstruction process after the 2009 L’Aquila earthquake. The following conclusions can be drawn:

1. FRP-strengthening was the technique used the most (34 and 56% of the buildings were subjected to repairs or global retrofitting, respectively) to conduct effective and rapid repairs and achieve an improved seismic performance.
2. The cost of implementing these solutions was about 94.4 €/m^2^ and 281.1 €/m^2^ for local strengthening and global retrofitting, respectively. These costs are lower than those associated with other strengthening techniques.
3. Strengthening the shear of unconfined beam-column joints was the technique used most, along with improving the infill-to-structure connections of perimetral infills using FRCM. This is because FRPs mainly work by increasing the displacement capacity of the structural system. Consequently, additional interventions are required to protect infills and partitions.
4. The actual direct costs associated with joint shear strengthening are an average of about 2051.5 €/member (38.4% RC) and 2939.2 €/member (55.0% RC) for systems designed within a local strengthening or a global retrofit strategy, respectively. The variation is due to differences in the quantity of members strengthened and the number of layers applied to the joint panels.
5. In the case of regular buildings with sufficient lateral stiffness, FRP systems can be effective seismic-strengthening solutions. Indeed, in this study, they enabled an increase in the safety index, ζ_E_, from an average of 0.3 to one of 0.7.

The results of this study may be very useful for researchers and practitioners involved in producing preliminary insights on the benefits and related costs associated with single-building or large-scale seismic-risk mitigation strategies for existing RC buildings. It should, however, be noted that the data collected in the study concern existing RC buildings typical of the municipality of L’Aquila, which was classified as a seismic zone in 1915. This should, therefore, be taken into account in any attempts to generalise the results to other regions.

## Figures and Tables

**Table 1:** Cost of FRP-strengthening interventions in a local-strengthening strategy (58 buildings).

FRP-Strengthening Intervention	Strengthened Buildings (n.)	Strengthened Buildings (%)	Mean n. of Layers	Direct Cost * (€/member)	Direct Cost ** (%RC/member)	Mean n. of Strengthened Members
Infill-frame connection	45	77.6	1.0	n.a.	n.a.	n.a.
Joint shear	39	67.2	1.6	2051.5	38.4	15.1
Beam flexure	14	24.1	1.2	572.1	9.5	5.9
Confinement	6	10.3	1.4	1305.6	32.5	13.3
Beam shear	6	10.3	1.0	1080.1	18.0	5.0
Column shear	1	1.7	1.0	1304.2	32.5	18
Column flexure	0	0.0	0.0	0.0	0.0	0.0

* These costs are discounted based on a rate calculated as the ratio of the cost of the FRP-strengthening solution at the time of the intervention and the current cost set out in the Abruzzo region’s price list. ** These were obtained by dividing the direct cost (€/member) by the member reconstruction cost, taken as 5344 €, 4008 € and 6012 € for the beam-column joints, columns, and beams, respectively.

**Table 2:** Costs of FRP-strengthening interventions in a global-strengthening strategy (16 buildings).

FRP-Strengthening Intervention	Strengthened Buildings (n.)	Strengthened Buildings (%)	Mean n. of Layers	Direct Cost * (€/member)	Direct Cost ** (%RC/member)	Mean n. of Strengthened Members
Infill-frame connection	8	50.0	1.0	n.a.	n.a.	n.a.
Joint shear	14	87.5	2.1	2939.2	55.0	37.4
Beam shear	5	31.2	1.5	1513.3	25.2	34.4
Column shear	5	31.2	2.0	2465.0	61.5	45.6
Beam flexure	3	18.7	1.3	1915.7	31.9	31.0
Confinement	2	12.5	1.0	1136.4	28.4	90.5
Column flexure	1	6.2	1.0	969.4	24.2	4.0

* These costs are discounted based on a discount rate calculated as the ratio of the cost of the FRP-strengthening solution at the time of the intervention and the current cost based on the Abruzzo region’s price list. ** These costs were obtained by dividing the direct cost (€/member) by the member reconstruction cost, taken as 5344 €, 4008 €, and 6012 € for the beam-column joints, columns and beams, respectively.

## Data Availability

The data presented in this study are available on request from the corresponding author.
